# Monocular deprivation during the critical period alters neuronal tuning and the composition of visual circuitry

**DOI:** 10.1371/journal.pbio.3002096

**Published:** 2023-04-21

**Authors:** Thomas C. Brown, Aaron W. McGee

**Affiliations:** Department of Anatomical Sciences and Neurobiology, School of Medicine; University of Louisville, Louisville, Kentucky, United States of America; Yale University, UNITED STATES

## Abstract

Abnormal visual experience during a developmental critical period degrades cortical responsiveness. Yet how experience-dependent plasticity alters the response properties of individual neurons and composition of visual circuitry is unclear. Here, we measured with calcium imaging in alert mice how monocular deprivation (MD) during the developmental critical period affects tuning for binocularity, orientation, and spatial frequency for neurons in primary visual cortex. MD of the contralateral eye did not uniformly shift ocular dominance (OD) of neurons towards the fellow ipsilateral eye but reduced the number of monocular contralateral neurons and increased the number of monocular ipsilateral neurons. MD also impaired matching of preferred orientation for binocular neurons and reduced the percentage of neurons responsive at most spatial frequencies for the deprived contralateral eye. Tracking the tuning properties for several hundred neurons before and after MD revealed that the shift in OD is complex and dynamic, with many previously monocular neurons becoming binocular and binocular neurons becoming monocular. Binocular neurons that became monocular were more likely to lose responsiveness to the deprived contralateral eye if they were better matched for orientation prior to deprivation. In addition, the composition of visual circuitry changed as population of neurons more responsive to the deprived eye were exchanged for neurons with tuning properties more similar to the network of responsive neurons altered by MD. Thus, plasticity during the critical period adapts to recent experience by both altering the tuning of responsive neurons and recruiting neurons with matching tuning properties.

## Abstract 2

How does abnormal visual during a developmentally critical period negatively impact cortical responsiveness? Calcium imaging in alert juvenile mice reveals how the receptive field-tuning properties of neurons in visual cortex change with abnormal vision during the critical period for ocular dominance plasticity.

## Introduction

The impact of experience on maturing neural circuitry is most profound during a **“**critical period**”** of heightened sensitivity in development [1]. How experience sculpts neural circuits has been studied extensively in the primary visual cortex (V1), where brief monocular deprivation (MD) during a critical period perturbs the response properties of neurons [2,3]. The outcome of visual plasticity evoked by MD during the critical period is an aggregate reduction in the magnitude of responses to the deprived eye that shifts ocular dominance (OD) towards the nondeprived fellow eye [4,5].

Calcium imaging in vivo has provided the opportunity to investigate how large populations of neurons in V1 respond to MD. Initial studies reported that layer (L) 2/3 excitatory neurons in the binocular zone of mouse V1 are almost exclusively binocular [6]. However, more recent imaging experiments with either calcium-sensitive dyes or the genetically encoded calcium sensor GCaMP6s have reported that a majority of neurons are monocular and predominantly respond to the contralateral eye [7–10]. The orientation and spatial frequency (SF) tuning of excitatory neurons in V1 in juvenile and adult mice have also been characterized separately in studies employing electrophysiology that isolated responses of individual neurons, as well as with calcium imaging. These studies measured the orientation tuning width and binocular matching for orientation, and tuning range and width for SF from naïve animals [6–9,11–15]. Interestingly, brief MD during the critical period reduces orientation tuning in cats but not mice [14,16].

In addition, the effects of MD on binocularity and orientation tuning in adult mice after the critical period has closed have been tracked over weeks for a relatively small number of neurons per mouse with calcium imaging [17]. This difficult longitudinal imaging study identified aspects of how MD alters the binocularity of a population of neurons pooled across adult mice, as well as how these neurons recover as a population following restoration of binocular vision following MD. This study also measured the preferred orientation for a few dozen neurons and determined that it was not affected by MD.

More recent work has demonstrated a surprising reorganization of the neuronal composition of visual circuitry in mice throughout life as poorly tuned binocular neurons are rendered monocular and sharply tuned monocular neurons convert to binocular neurons [9]. This process is disrupted by eliminating visual experience with dark exposure during the critical period, a manipulation that does not engage OD plasticity [18]. Here, we employed calcium imaging to determine how abnormal visual experience during the critical period caused by MD of the contralateral eye alters the population of neurons active in visual circuitry and affects neuronal tuning for binocularity, orientation, and SF.

## Results

We implanted cranial windows at postnatal day (P) 24 to 30 in mice expressing GCaMP6s in excitatory neurons of the forebrain [19,20] (S1A Fig and Table 1). The next day, we identified the binocular zone of visual cortex by wide-field calcium imaging [20] (S1B Fig). Thereafter, we measured calcium responses in alert mice in response to a battery of sinusoidal gratings across a range of orientation (0 to 180 degrees, 30 degree intervals) and SF (0.02 to 0.48 cycles per degree (cpd), intervals spaced at half octaves (log(1.5)) (S1C and S1D Fig). Visual stimuli were presented independently to each eye.

**Table 1:** Reagents and resources employed in the study.

REAGENT or RESOURCE	SOURCE	IDENTIFIER
**Deposited data**		
Calculated tuning properties for all neurons		N/A
**Experimental models: Organisms/strains**		
Mouse: B6;DBA-Tg(tedO-GCaMP6s)2Niell/j	The Jackson Laboratory	RRID: ISMR_ JAX: 024742
Mouse: B6;Cg-Tg(Camk2a-tTA)1Mmay/DboJ	The Jackson Laboratory	RRID: ISMR_ JAX: 007004
**Software and algorithms**		
MATLAB	Mathworks	https://www.mathworks.com/
Processing2	Processing	https://processing.org/

To measure the tuning properties in L2/3 and L4 in V1, first, we identified neuronal soma as regions of interest (ROIs) and then determined the z-score and inferred spike rate (ISR) for each ROI from the normalized change in fluorescence (dF/F) for each combination of orientation and SF (S1E–S1I Fig) [21,22]. We identified visually responsive neurons from these ROIs based on the delay from stimulus onset (in imaging frames), the signal-to-noise ratio (SNR) [9], and the number of responses (spike ratio (SR)) at the optimal delay (S1J–S1O Fig). The preferred orientation and SF were calculated from the row (orientation) and column (SF) corresponding to the maximal ISR at the optimal delay (frame number). In total, we evaluated calcium responses from 5,557 neurons in this experiment, 2,723 (approximately 50%) of which met our inclusion criteria for visually responsive neurons.

**Fig 1:**
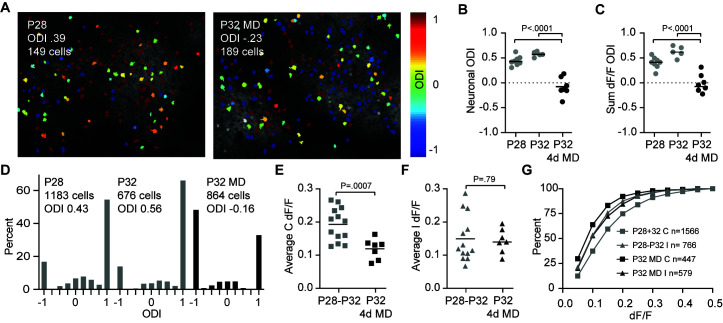
Measuring OD plasticity with calcium imaging at cellular resolution. (**A**) Imaging fields for P28 (left) and P32 after 4 days of MD of the contralateral eye (right). Neurons are color-coded to their ODI score. 1 corresponds to monocular contralateral neurons. −1 corresponds to monocular ipsilateral neurons. (**B**) Average neuronal ODI scores for nondeprived P28–P32 mice (mean ODI = 0.43, *n =* 8 mice), P32 mice (mean ODI .57, *n* = 5 mice), and P32 mice after 4 days of MD (mean ODI = −0.09, *n =* 7 mice). P32 mice after 4 days of MD possess significantly different ODI values than both P28 and P32 nondeprived mice. (1-way ANOVA) (**C**) Sum ODI scores for the same mice as in panel B. P28 (mean ODI = 0.39, *n =* 8 mice), P32 (mean ODI = 0.61, *n* = 5 mice), and P32MD (mean ODI = −0.02, *n* = 7 mice). P32 mice after 4 days of MD possess significantly different ODI values than both P28 and P32 nondeprived mice. (1-way ANOVA). (**D**) Histogram of ODI scores for nondeprived P28 (left), nondeprived P32 mice (middle), and P32 mice following 4 days (4d) of MD (right) from panel B. (**E**, **F**) Mean normalized fluorescence (delta F over F, dF/F) values for neurons responding to the contralateral eye (C) and ipsilateral eye (I) for each nondeprived mouse (P28–P32, *n =* 13) and mice after 4 days of MD (P32 4d MD, *n =* 7) (Welch’s *t* test). (**G**) Cumulative histogram of the distribution of normalized fluorescence responses (dF/F) from P28–P32 mice for visual stimuli provided to the contralateral eye (C, *n =* 1,566 neurons) or the ipsilateral eye (I, *n* = 766 neurons), as well as P32 mice after 4 days of MD of the contralateral eye (C, *n* = 447 neurons; I, *n* = 579 neurons). https://data.mendeley.com/datasets/3yt5kpzw6d. MD, monocular deprivation; OD, ocular dominance; ODI, ocular dominance index; P, postnatal day.

We calculated the ocular dominance index (ODI) for each neuron from the dF/F for the visual stimulus capturing the preferred orientation and SF for nondeprived mice at P28 and P32, as well as mice at P32 after 4 days of MD of the contralateral eye (Fig 1A). A majority of neurons responsive to the contralateral eye did not display a significant response when the visual stimuli were presented to the ipsilateral eye, resulting in an ODI score of 1. Neurons that only displayed a significant response to the ipsilateral eye were assigned an ODI score of −1. Nondeprived mice were more responsive to visual stimuli presented to the contralateral eye, with higher average ODI values per mouse. By comparison, mice receiving 4-day MD displayed lower average ODI values (*P* < 0.0001) (Fig 1B).

In addition, to examine the possibility that the mean ODI per mouse was biased by a population of weakly responding predominantly monocular neurons, we also calculated an ODI score from the sum of the dF/F values for stimuli presented to the contralateral eye and ipsilateral eye from all visually responsive neurons for each mouse (Fig 1C). This metric sums the response strength across neurons to evaluate OD and is analogous to the calculation for ODI employed for optical imaging of intrinsic signals [23]. Contralateral bias was similarly reduced in mice following 4 days of MD as measured with this approach (*P* < 0.0001). Thus, OD plasticity can be measured by calcium imaging either as the average ODI of individual neurons per mouse or as the ODI calculated from the sum of dF/F for each eye for all responsive neurons per mouse.

Histograms of the distribution of ODI values for neurons from nondeprived mice at both P28 and P32 reveal the typical contralateral bias of neuronal responses in V1 (Fig 1D). By comparison, neurons from mice receiving 4 days of MD of the contralateral eye initiated at P28 displayed significant shifts in OD histograms. This difference was driven in part by an increased percentage of ipsilateral monocular neurons (Fig 1D).

Experiments employing visually evoked potential**s (**VEPs**)** or optical imaging of intrinsic signals report that depression of responsiveness to the closed contralateral eye is the principal mechanism of OD plasticity during the critical period [4,5]. To probe how MD alters responsiveness to each eye at neuronal resolution, we compared the dF/F responses for visual stimuli presented to either the contralateral or ipsilateral eye for nondeprived mice and those receiving 4-day MD. Consistent with these preceding measurements of aggregate neuronal activity, deprivation reduced the average response amplitude for the contralateral eye per mouse (*P* = 0.0007) but did not affect responses to the fellow ipsilateral eye (*P* = 0.79) (Fig 1E and 1F). This reduction of responses for the contralateral eye was evident across a range of response strengths (Fig 1G).

**Fig 2:**
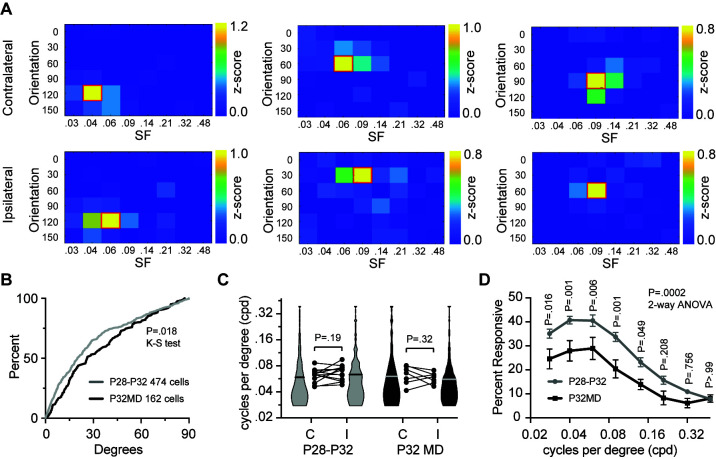
MD during the critical period reduces binocular orientation matching and the number of responding neurons across most spatial frequencies. **(A)** Examples of heat maps for responses of the contralateral eye (top) and ipsilateral eye (bottom) across orientation and SF for binocular neurons with a difference of orientation preference of 5 degrees (left), 16 degrees (middle), and 40 degrees (right) from a naïve mouse (left) and mice after 4 days of MD (middle, right). **(B)** Cumulative distribution of difference in preferred orientation for the 2 eyes by binocular neurons for nondeprived mice (*n =* 474, median = 20 degrees) and after 4 days of MD of the contralateral eye (*n* = 162, median = 26 degrees) (*P* = 0.018; Kolmogorov–Smirnov test of cumulative distribution (KS test)). **(C)** The mean preferred SF for neurons responding to the contralateral eye (C) and ipsilateral eye (I) for each nondeprived mouse (P28–P32, *n* = 13) and mice after 4 days of MD (P32MD, *n* = 7). The distribution and median for the population of neurons are presented as violin plots with the median indicated by a horizontal bar (P28–P32, C = 1,566 neurons, I = 766 neurons; P32MD, C = 447 neurons, I = 579 neurons). There is no statistical difference in the mean preferred SF for the C and I eye per mouse for either nondeprived mice or following 4 days of MD (paired *t* test). **(D)** The percent of visually responsive neurons per mouse that displayed a significant response to each SF at any orientation (2-way ANOVA with Sidak’s multiple comparison test for 8 comparisons). https://data.mendeley.com/datasets/3yt5kpzw6d. MD, monocular deprivation; SF, spatial frequency.

MD did not alter the distribution of preferred orientation but reduced binocular matching of preferred orientation (Fig 2A and 2B). The matching of orientation preference of P28-P32 binocular neurons for nondeprived mice was significantly greater than P32 mice following MD (*P* = 0.018; K-S test) (Fig 2B). These results confirm findings with electrophysiologic recordings [14]. The distribution of preferred SFs for neurons were similar for the contralateral and ipsilateral eye between nondeprived mice and following MD (Fig 2C). The average preferred SF per mouse was also similar for neurons responsive to the contralateral and ipsilateral eye for both P28-P32 nondeprived mice and mice receiving 4-day MD (Fig 2C). However, despite normal distribution of preferred SF, the percentage of visually responsive neurons for the contralateral eye that displayed significant responses for each SF was markedly reduced in P32MD mice (*P* < 0.0002, 2-way ANOVA) (Fig 2D). This reduction of responsiveness in the population of neurons correlates with the lower range VEPs measured after MD and may contribute to lower acuity measured with behavioral assays [4].

**Fig 3:**
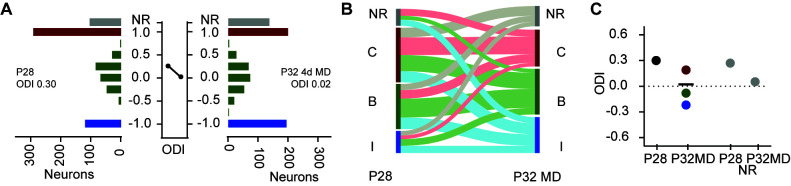
Tracking neurons longitudinally reveals an exchange of monocular and binocular neurons as well as neurons active in visual circuitry during OD plasticity. **(A)** Histogram of ODI values at P28 (left) and at P32 after 4 days of MD of the contralateral eye (right) for visually responsive neurons, nonresponsive (NR) neurons at P28 that were visually responsive at P32, and visually responsive neurons that became nonresponsive (NR) at P32, for 6 mice receiving 4 days of MD starting at P28. A line between the 2 histograms connects points that indicate the mean ODI of the population of visually responsive neurons at P28 and P32MD, respectively. **(B)** Sankey diagram of the stability and interconversion between P28 and P32MD for neurons that were nonresponsive (NR, grey), monocular contralateral (C, red), binocular (B, green) and monocular ipsilateral (I, blue) for the neurons presented in panels A. (**C**) The mean ODI for all neurons by category presented in panels A and D. The mean ODI of neurons that were visually responsive at P28 (black) and the mean of ODI at P32 of these neurons categorized as monocular contralateral (red), binocular (green), and monocular ipsilateral (blue) at P28. The black horizonal line indicates the mean ODI of all neurons visually responsive at P32 that were also visually responsive at P28. In addition, the mean ODI of neurons at P28, which were nonresponsive (NR) at P32, and the mean ODI of neurons at P32, which were nonresponsive at P28, are plotted to the right. https://data.mendeley.com/datasets/3yt5kpzw6d. MD, monocular deprivation; OD, ocular dominance; ODI, ocular dominance index; NR, nonresponsive.

To determine how MD during the critical period alters tuning properties of individual neurons, we measured the responses of the same neurons in L2/3 on P28 and P32 after MD of the contralateral eye (Figs 3 and S3). We determined the location of the same population of neurons for repeated calcium imaging by depth of focus combined with the positions of neurons with high baseline fluorescence and features of the microvasculature as reference points [9,24,25]. Given the size of neuronal soma, the magnitude of the dF/F calcium signal is resistant to minor differences in focal depth [9]. However, to account for potential differences in the angle of the focal plane associated with repeatedly positioning the mouse for imaging, and to provide additional certitude that the same population of neurons was imaged in both sessions, we confined our analysis to the imaging plane circumscribed by a perimeter of neurons with tuning properties that did not change between imaging sessions (S3 Fig). These neurons possessed preferred orientation that varied by less than 30 degrees (median 4 degrees, mean 7 degrees) and preferred SF that deviated by less than an octave from P28 to P32 (median 0.23 octaves, mean 0.25 octaves) (S3 Fig).

Neurons imaged at P28 displayed significant alterations to binocularity at P32 after 4 days of MD of the contralateral eye. The distribution of ODI values shifted towards the nondeprived eye (P28, 656 neurons, mean ODI 0.30; P32, 639 neurons, mean ODI 0.02) (Fig 3A). Consistent with the OD histograms for mice imaged only after MD of the contralateral eye (Fig 1D), OD plasticity decreased the ratio of monocular contralateral neurons to monocular ipsilateral neurons. A Sankey plot illustrates the complex interconversions of the tuning for binocularity for neurons between P28 and P32 following MD (Fig 3B). At P28, we identified 294 monocular contralateral neurons, 243 binocular neurons, and 120 monocular ipsilateral neurons. At P32, the number of monocular contralateral neurons decreased to 198 neurons, while the number of monocular ipsilateral neurons increased to 194 neurons. Nearly half of monocular contralateral neurons at P28 gained responsiveness to the nondeprived eye following MD. The average ODI of the population of neurons that were either monocular contralateral, binocular, and monocular ipsilateral at P28 shifted to a distribution of ODI values near zero after 4 days of MD (Fig 3C). In addition, neurons that were nonresponsive at P28 but visually responsive at P32 displayed lower average ODI values (ODI = 0.05) than neurons visually responsive at P28 but nonresponsive at P32 (ODI = 0.27). Thus, OD plasticity both converts monocular contralateral neurons into binocular neurons and monocular ipsilateral neurons, as well as recruits into visual circuitry nonresponsive neurons that adopt a similar distribution of ODI values (Fig 3B and 3C).

**Fig 4:**
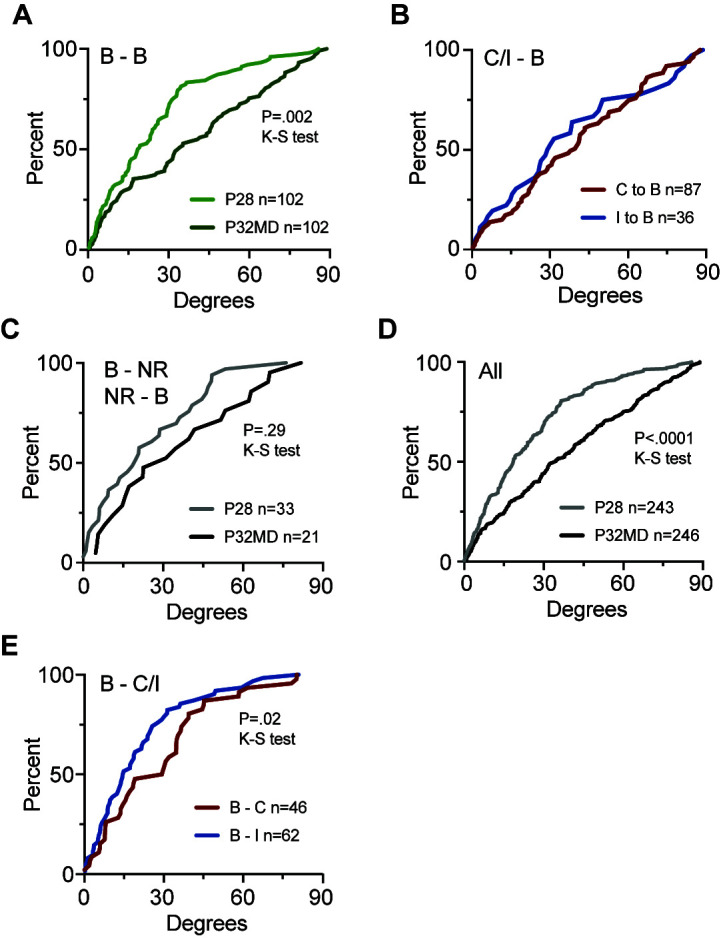
MD during the critical period degrades binocular orientation matching. **(A)** Cumulative distribution of difference in preferred orientation for the 2 eyes by neurons that were binocular (B—B) both at P28 (*n* = 102, median = 19 degrees) and at P32 after 4 days of MD (*n* = 102, median = 34 degrees). (*P* < 0.0001; KS test). **(B)** Cumulative distribution of difference in preferred orientation for neurons that were either contralateral monocular (C) or ipsilateral monocular (I) at P28 and binocular (B) at P32 following MD (C-B, *n* = 87, median 39 degrees; I-B, *n* = 36, 30 degrees) (**C**) Cumulative distribution of difference in preferred orientation for neurons that were binocular at P28 but nonresponsive at P32 (B-NR, *n* = 33, median = 19 degrees) and at P32 after 4 days of MD (NR-B, *n* = 102, median = 34 degrees). (*P* < 0.0001; KS test). **(D)** Cumulative distribution of difference in preferred orientation for the 2 eyes by all binocular neurons (All) at P28 (*n =* 243, median = 19 degrees) and P32 after 4 days of MD (*n* = 246, median = 34 degrees). (*P* < 0.0001; KS test). **(E)** The cumulative distribution of difference in preferred orientation (degrees) for neurons that were binocular at P28 but either lost responsiveness to the fellow ipsilateral eye to become contralateral monocular (B-C, red, *n* = 46 neurons; median 29 degrees) or lost responsiveness to the deprived contralateral eye to become ipsilateral monocular (B-I, blue, *n* = 62 neurons; median 15 degrees). These distributions are statistically different (*P* = 0.02, KS test). https://data.mendeley.com/datasets/3yt5kpzw6d. MD, monocular deprivation.

OD plasticity also disrupted binocular matching of preferred orientation. Matching degraded for neurons that were binocular both at P28 (median 19 degrees) and P32 after MD (median 33 degrees) (Fig 4A). Neurons that were contralateral monocular or ipsilateral monocular at P28 but gained responsiveness to the other eye to become binocular at P32 also displayed binocular matching of preferred orientation worse than binocular neurons at P28 (contralateral monocular to binocular, median 39 degrees; ipsilateral monocular to binocular, median 30 degrees) (Fig 4B). Neurons that were visually responsive at P28 but nonresponsive after MD displayed better matching of preferred orientation (median 20 degrees) than neurons nonresponsive at P28 but visually responsive at P32 (median 31 degrees) (Fig 4C). However, due to the smaller sample size (33 and 21 neurons, respectively), this comparison did not reach statistical significance (*P* = 0.29), although the medians for the 2 groups were similar to the total populations of binocular neurons at the 2 time points (P28, 18 degrees, P32MD 34 degrees) (Fig 4D). Interestingly, binocular neurons at P28 that lost responsiveness to ipsilateral eye P32 following MD had worse binocular matching for preferred orientation (*n =* 46, median 29 degrees) than neurons that lost responsiveness to the contralateral eye (*n* = 62, median 15 degrees) (*P* = 0.02, KS test) (Fig 4E).

To explore the mechanism for reduced matching of preferred orientation, we compared the difference in orientation preference between the 2 eyes to the stability of preferred orientation for the contralateral eye or ipsilateral eye for binocular neurons (S4A–S4C Fig). These comparisons did not reveal any evident correlation. In addition, we performed a similar comparison for the binocular matching of neurons that converted from monocular contralateral or monocular ipsilateral at P28 to binocular after 4 days of MD. These comparisons also did not indicate an evident correlation between binocular matching and the stability of orientation preference for contralateral monocular neurons and ipsilateral monocular neurons that converted to binocular (S4D and S4E Fig). Likewise, the stability of orientation preference for binocular neurons that converted to monocular was similar to neurons that remained monocular after MD (S4F Fig).

## Discussion

The effects of MD on binocularity in visual cortex were first characterized cats and primates from **“**single units**”** quantified by auditory discrimination [26–28]. These seminal studies revealed the presence of a **“**critical period**”** during development when visual circuitry is most sensitive to the quality of experience. Experiments of similar design were later performed with rodents [29–31]. Units were typically only examined for OD because quantifying additional tuning properties manually was arduous.

Subsequent studies in the rodents have employed techniques to study OD plasticity that report aggregate neuronal activity, such as VEPs, optical imaging of intrinsic signals, and multi-unit electrophysiologic recordings [4,23,32]. VEPs reflect a combination of subthreshold synaptic activity and spiking activity from multiple neurons [33]. Optical imaging of intrinsic signals is an indirect measurement of neural activity coupled to a hemodynamic response [34,35].

Although these techniques are not suitable for measuring tuning properties at neuronal resolution, they have been instrumental for elucidating several characteristics of OD plasticity during the critical period that discriminate it from the less robust plasticity remaining in the adult brain after the critical period closes [36]. OD plasticity during the critical period is predominantly driven by an overall depression of responsiveness to the deprived eye [4,5]. Shifts in binocularity are also preceded by intracortical disinhibition and are insensitive to benzodiazepines and barbiturates. In contrast, OD plasticity in adults is smaller in magnitude, results from potentiation of responses to the fellow eye, is not associated with disinhibition, and is suppressed by benzodiazepines and barbiturates [5,37–41]. A resulting model for OD plasticity during the critical period is that occluding vision through one eye attenuates sensory input to lower the activity of neurons in V1, which, in turn, drives intracortical disinhibition that facilitates competitive synaptic plasticity to alter binocularity [37].

Classic studies employing auditory discrimination of electrophysiologic recordings and more recent studies measuring multi-unit activity with higher impedance electrodes report that the majority of responses in mouse V1 are binocular [31,32,42–44]. A limitation of these recordings is that they represent the activity from small populations of neurons at each recording location rather than from a single neuron. By comparison, calcium imaging provides several key advantages for measuring the tuning properties of individual neurons, particularly with GCaMP6s that can reliably detect single action potentials in neuronal somata [45]. First, calcium imaging provides certitude that responses originate from individual neurons [45]. Second, sampling is greatly improved as large populations of neurons can be imaged simultaneously [9,46,47] Third, imaging can be performed longitudinally to monitor the stability of tuning properties [9,48,49]. Multiple research groups have exploited these advantages to examine the fraction of binocular neurons in mouse V1 with calcium imaging and GCaMP6s; each report that the majority of neurons in L2/3 of V1 are predominantly monocular while a smaller fraction of neurons are binocular [7–10].

To understand how OD plasticity during the critical period operates at neuronal resolution, first, we measured the binocularity, response strength, orientation tuning, and SF tuning, for thousands of neurons with calcium imaging and GCaMP6s. Then, we tracked how MD altered these properties for hundreds of neurons with repeated calcium imaging. MD shifts OD towards the nondeprived eye by reducing both the number and strength of neuronal responses to the deprived contralateral eye while increasing the number but not the strength of neuronal responses to the fellow ipsilateral eye. These findings explain the reduced amplitude of response to the deprived contralateral eye measured with VEPs and optical imaging of intrinsic signals [4,5]. Interestingly, the principal mechanism of OD plasticity was not a uniform shift in OD, but a reduction in the ratio of contralateral monocular neurons to ipsilateral monocular neurons with smaller effects on the population of binocular neurons.

We observe that MD during the critical period did not affect orientation tuning but impaired matching of orientation preference for binocular neurons. These findings are consistent with a previous study measuring binocular matching of units isolated from electrophysiologic recordings [14]. MD also did not appear to alter the distribution of SF preference for neurons. However, it significantly reduced the percentage of the neurons responsive to the deprived eye across a range SFs. This appears to be a consequence of the overall depression of responses to the contralateral eye and may inform interpretation of VEPs across SF that have been employed to estimate acuity [18,50].

A preceding calcium imaging study examined the effects of a week of MD in adult mice on binocularity and orientation preference [17]. They reported that adult OD plasticity is mediated by a shift in the relative responsiveness of a predominant population of binocular neurons towards the nondeprived eye, a reduction in the strength of response to the contralateral (closed) eye, and an increase in the strength of responses to the ipsilateral (nondeprived) eye. MD of adult mice did not affect orientation preference. By comparison, measurements of OD plasticity with either VEPs and optical imaging report that adult OD plasticity is mediated by strengthening of responses for the ipsilateral eye, not depression of responses to the contralateral eye [5,36,41]. Some of the variability in neuronal binocularity evident in these longitudinal experiments was likely a consequence of the interconversion neurons between binocular and monocular for either eye [9]. Changes to the composition of binocular circuitry are more prominent during the critical period but also substantial in adult mice [9].

Tracking the tuning properties of neurons before and after MD revealed that abnormal vision engages the synaptic mechanisms that both alter neuronal tuning for binocularity and exchange neurons active in visual circuitry. MD converted a fraction contralateral monocular neurons to binocular neurons and rendered a similar fraction of binocular neurons monocular and responsive to the contralateral and ipsilateral eye in near equal proportions. This reorganization of visual circuitry reduced the ratio of monocular neurons responsive to the deprived contralateral eye versus the fellow ipsilateral eye and thereby decreased the contralateral bias that is characteristic of mouse visual cortex [23,42,50]. These alterations in the binocularity of neurons were accompanied by an exchange of neurons active in visual circuitry that matched the altered binocularity of neurons that were visually responsive at both P28 and at P32 after 4 days of MD. We propose that altering the tuning of responsive neurons, recruiting neurons with matching tuning properties, and a depression of the strength of responses to the contralateral eye are the neuronal basis for OD plasticity during the critical period.

## Methods

### Materials availability

This study did not generate new unique reagents.

### Experimental model and subject details

All procedures were approved by University of Louisville Institutional Animal Care and Use Committee (IACUC) protocol 22105 and were in accord with guidelines set by the US National Institutes of Health. Mice were anesthetized by isoflurane inhalation and killed by carbon dioxide asphyxiation or cervical dislocation following deep anesthesia in accordance with approved protocols. Mice were housed in groups of 5 or fewer per cage in a 12/12 light–dark cycle. Animals were naive subjects with no prior history of participation in research studies. A total of 26 mice, both male (13) and female (13) were used in this study. The following male and female mice are represented in the following groups: P28-P32 nondeprived mice, 7 males and 6 females; P32 4MD, 3 males and 4 females; P28-P32 4MD repeat imaging, 3 males and 3 females.

### Mice

Imaging was performed on mice expressing GCaMP6S in excitatory neurons in forebrain. The *CaMKII-tTA* (stock no. 007004) and *TRE-GCaMP6s* (stock no. 024742) transgenic mouse lines were obtained from Jackson Labs [20,51]. Mice were genotyped with primer sets suggested by Jackson Labs.

## Methods

### Cranial window surgeries

All epifluorescent and 2-photon imaging experiments were performed though a cranial window. In brief, mice were administered carprofen (5 mg/kg) and buprenorpine (0.1 mg/kg) for analgesia and anesthetized with isoflurane (4% induction, 1% to 2% maintenance). The scalp was shaved and mice were mounted on a stereotaxic frame with palate bar and their body temperature maintained at 37°C with a heat pad controlled by feedback from a rectal thermometer (Physitemp). The scalp was resected, the connective tissue removed from the skull, and an aluminum headbar affixed with C&B metabond (Parkell). A circular region of bone 3 mm in diameter centered over left visual cortex was removed using a high-speed drill (Foredom). Care was taken to not perturb the dura. A sterile 3 mm circular glass coverslip was sealed to the surrounding skull with cyanoacrylate (Pacer Technology) and dental acrylic (ortho-jet, Lang Dental). The remaining exposed skull likewise sealed with cyanoacrylate and dental acrylic. Mice recovered on a heating pad. Mice were left to recover for at least 2 days prior to 2-photon imaging.

### Wide-field calcium imaging

After implantation of the cranial window and before 2-photon imaging, the binocular zone of visual cortex was identified with wide-field calcium imaging similar to our method for optical imaging of intrinsic signals [52]. In brief, mice were anesthetized with isoflurane (4% induction), provided a low dose of the sedative chlorprothixene (0.5 mg/kg IP; C1761, Sigma) and secured by the aluminum headbar. The eyes were lubricated with a thin layer of ophthalmic ointment (Puralube, Dechra Pharmaceuticals). Body temperature was maintained at 37°C with heating pad regulated by a rectal thermometer (TCAT-2LV, Physitemp). Visual stimulus was provided through custom-written software (MATLAB, Mathworks). A monitor was placed 25 cm directly in front of the animal and subtended +40 to −40 degrees of visual space in the vertical axis. A horizonal white bar (2 degrees high and 20 degrees wide) centered on the zero-degree azimuth drifted from the top to bottom of the monitor with a period of 8 seconds. The stimulus was repeated 60 times. Cortex was illuminated with blue light (475 ± 30 nm) (475/35, Semrock) from a stable light source (intralux dc-1100, Volpi). Fluorescence was captured utilizing a green filter (HQ620/20) attached to a tandem lens (50 mm lens, computar) and camera (Manta G-1236B, Allied Vision). The imaging plane was defocused to approximately 200 μm below the pia. Images were captured at 10 Hz as images of 1,024 × 1,024 pixels and 12-bit depth. Images were binned spatially 4 × 4 before the magnitude of the response at the stimulus frequency (0.125 Hz) was measured by Fourier analysis.

### Visual stimulus and 2-photon calcium imaging

Visual stimulus presentation and image acquisition were both performed according to published methods with minor modifications [9,53]. In brief, a battery of static sinusoidal gratings was generated in real time with custom software (Processing, MATLAB). Stimulus presentation was synchronized to the imaging data by time stamping the presentation of each visual stimulus to the image acquisition frame number a transistor–transistor logic (TTL) pulse generated with an Arduino at each stimulus transition. Orientation was sampled at equal intervals of 30 degrees from 0 to 150 degrees (6 orientations). SF was sampled in 8 steps on a logarithmic scale at half-octaves from 0.028 to 0.48 cpd. An isoluminant grey screen was included (blank) was provided as a ninth step in the SF sampling as a control. Spatial phase was equally sampled at 45-degree intervals from 0 to 315 degrees for each combination of orientation and SF. Gratings with random combinations of orientation, SF, and spatial phase were presented at a rate of 4 Hz on a monitor with a refresh rate of 60Hz. Imaging sessions were 10 minutes (2,400 presentations in total). Consequently, each combination of orientation and SF was presented 40 times on average (range 29 to 56). The monitor was centered on the zero azimuth and elevation 35 cm away from the mouse and subtended 45 (vertical) by 80 degrees (horizontal) of visual space.

Imaging was performed with a resonant scanning 2-photon microscope controlled by Scanbox image acquisition and analysis software (Neurolabware). The objective lens was fixed at vertical for all experiments. Fluorescence excitation was provided by a tunable wavelength infrared laser (Ultra II, Coherent) at 920 nm. Images were collected through a 16× water-immersion objected (Nikon, 0.8 NA). Images (512 × 796 pixels, 520 × 740 μm) were captured at 15.5 Hz at depths between 150 and 400 μm. Eye movements and changes in pupil size were recorded using a Dalsa Genie M1280 camera (Teledyne Dalsa) fitted with 50 mm 1.8 lens (Computar) and 800 nm long-pass filter (Edmunds Optics). Imaging was performed on alert mice positioned on a spherical treadmill by the aluminum head bar affixed to the skull. The visual stimulus was presented to each eye separately by covering the fellow eye with a small custom occluder.

### Image processing

Image processing was performed as described previously with minor modifications [9]. In summary, imaging series for each eye were motion corrected with the SbxAlign tool. ROIs corresponding to excitatory neurons were selected manually with the SbxSegment tool following computation of pixel-wise correlation of fluorescence changes over time from 350 evenly spaced frames (approximately 4%). ROIs for each experiment were determined by correlated pixels the size similar to that of a neuronal soma. The fluorescence signal for each ROI and the surrounding neuropil were extracted from this segmentation map.

### Image analysis to identify visually responsive neurons and calculate their tuning properties

Image analysis was performed as described previously with minor modifications [9]. The fluorescence signal for each neuron was extracted by computing the mean of the calcium fluorescence within each ROI and subtracting the median fluorescence from the surrounding perimeter of neuropil [9,21]. An ISR was estimated from adjusted fluorescence signal with the Vanilla algorithm [22]. A reverse correlation of the ISR to stimulus onset was used to calculate the preferred stimuli [9,21,53,54]. Neurons that satisfied 3 criteria were categorized as visually responsive: (1) the ISR was highest with the optimal delay of 4 to 9 frames following stimulus onset. This delay was determined empirically for this transgenic GCaMP6s mouse [9]; (2) the SNR was greater than the 75th percentile of spontaneously active neurons. The signal is the mean of the spiking standard deviation at the optical delay between 4 and 9 frames after stimulus onset and the noise this value at frames −2 to 0 before the stimulus onset or 15 to 18 after it [9,53]; (3) and the percent of responses to the preferred stimulus was greater than the 75th percentile of spontaneously active neurons (see S1 Fig). Visual responsiveness for every neuron was determined independently for each eye. The visual stimulus capturing the preferred orientation and SF was the determined from the matrix of all orientations and SFs presented as the combination with highest average ISR.

The preferred orientation for each neuron was calculated as:

$$orientation=\frac{\arctan\left(\sum_{n}O_{n}*e^{i*2*\pi *\theta_{n}/180}\right)}{2}$$

O_n_ is a 1 × 6 array of the mean z-scores associated with the calculation of the ISR at orientations Q_n_ (0 to 150 degrees, spaced every 30 degrees). Orientation calculated with this formula is in radians and was converted to degrees. The tuning width was the full width at half-maximum of the preferred orientation.

The preferred SF for each neuron was calculated as:

$$SF=10^{\frac{\sum_{k}{Sf}_{\kappa }*{log}_{10^{\omega_{\kappa }}}}{\sum_{\kappa }{Sf}_{\kappa }}}$$

Sf_k_ is a 1 × 8 array of the mean z-scores at SFs w_k_ (8 equal steps on a logarithmic scale from 0.028 to 0.481 cpd). Tails of the distribution were clipped at 25% of the peak response. The tuning width was the full width at half-maximum of the preferred SF in octaves. The percent visually responsive neurons with significant responses at each SF was determined by comparing the distribution of ISR values at each SF versus the stimulus blank with a KW-test with Dunn’s correction for 8 comparisons. Neurons with *P* < 0.01 for a given SF were considered significant responses at that SF [7].

### Binocular matching of preferred orientation

Binocular matching was measured as the absolute difference in the preferred orientation calculated for visual stimuli presented to the contralateral eye and ipsilateral eye along the 180 degree cycle [14,55,56].

### Ocular dominance index (ODI)

Neuronal ODI was calculated as (C − I) / (C + I), where C and I are the mean normalized change in fluorescence (dF/F) for the preferred visual stimulus for the contralateral eye and ipsilateral eye, respectively. In cases where neurons displayed no significant response to visual stimuli provided to one eye, they were considered monocular for the other eye and assigned ODI values of 1 (contralateral) and −1 (ipsilateral) [7].

Summed ODI was calculated by summing the dF/F for the preferred visual stimulus for the all neurons visually responsive to the contralateral eye (C) and ipsilateral eye (I) for each mouse, respectively. The summed ODI per mouse was then calculated as (C − I) / (C + I) for each mouse.

### Longitudinal imaging of neurons before and after MD

The same imaging plane was identified for the second imaging experiment by using the reference image of the first experiment. The location in the neuropil was achieved predominantly by coordinating the depth from pial surface and the position of small blood vessels (S3A Fig). Fine adjustment of position was performed by matching responsive neurons evident in the reference image from the first experiment while presenting the visual stimulus.

Each imaging session was segmented independently, and every ROI was assigned a unique number. No geometric transformations were performed to match segmentation masks for ROIs from the 2 imaging sessions. The segmentation masks for the 2 imaging sessions were then compared and ROIs with at least 50% overlap were considered the same neuron. A perimeter of neurons with overlapping ROIs and tuning properties that did not change between imaging sessions (a difference in orientation preference of less than 30 degrees and SF preference of less than an octave) defined the matched imaging plane. To determine the SNR values of lost neurons at P32 and gained neurons at P28, the segmentation masks were exchanged between time points and the SNR from ROIs for the corresponding neurons at the other time point were calculated.

### Monocular deprivation

The right eye was sutured closed with a single mattress suture with 6–0 Prolene monofilament (Ethicon 8709) [32]. Prior to imaging, mice were briefly (<5 minutes) anesthetized with isoflurane (4% induction, 1% to 2% maintenance), the suture removed with Vannas scissors (Fine Science Tools). The eye was flushed with sterile saline and examined for corneal abrasions with a stereomicroscope. The mouse was then immediately head-fixed for imaging and allowed to recover for no less than 45 minutes. The occluder was positioned over one eye as soon as the mouse was head-fixed and occluded one of the eyes at all times. At no point during the experiment were mice permitted unobstructed binocular vision.

### Statistics

No statistical methods were used to predetermine sample size. All statistical analyses were done using Prism 8 software (GraphPad Software). All data sets were examined for normality, and 2-tail *t* tests were only employed for data with normal distributions. The nonparametric Mann–Whitney (MW) test for pairwise comparisons, the Kolmogorov–Smirnov test of cumulative distribution (K-S test), and Kruskal–Wallis (KW) test with Dunn’s correction for multiple comparisons were employed for data that did not conform to a normal distribution.

## Supporting information

S1 Fig Example of cranial window, identification of the binocular zone of V1, cell segmentation, signal extraction, deconvolution, and identification of visually responsive neurons. **(A**) A cranial window 3 mm in diameter implanted over visual cortex. Scale bar = 1 cm. Rostral–caudal and medial–lateral axes are indicated at upper left. **(B)** Wide-field calcium imaging of neuronal activity in response to a horizonal bar 30 degrees wide and 2 degrees high drifting down at 10 degrees per second. The white rectangle indicates the imaging field in (C) and (D). Scale bar = 1 cm. **(C)** Schematic of the visual stimulus. Sinusoidal gratings at 30 degrees intervals in orientation and between 0.028 and 0.48 cpd in SF spaced at half octaves (log(1.5)) as well as an isoluminant grey screen are presented in random order at 4 Hz for 10 minutes. Each combination of orientation and SF is presented 40 times on average (range 29–56). **(D)** Schematic of the setup for calcium imaging of alert mice. The monitor is positioned 35 cm away from the mouse centered at the zero azimuth and elevation. A mouse is alert, head-fixed, and freely moving on a styrofoam ball floating on column of air. A camera records pupil diameter. **(E)** An example reference image of imaging plane in the binocular zone of visual cortex. Imaging field is 750 μm × 500 μm. Scale bar = 100 μm. **(F**) Segmented neurons from the imaging field in (E). White circles correspond to ROIs identified manually. A total of 215 neuronal ROIs are segmented in this field. Scale bar = 100 μm. **(G)** Representative calcium trace (black line, top) and ISR (red line, middle) from an example neuron in the imaging field in (F). The timing of presentations of the preferred stimulus (90 degrees, 0.06 cpd) during the experiment (black vertical lines, bottom). Images are collected at 15.5 Hz. The scale bar represents 65 seconds, 1,000 frames, and 258 visual stimuli (grey horizonal bar, top right). Entire trace represents 10 minutes during which 2,400 gratings were presented from 56 combinations (6 orientations and a grey screen for each of 8 SFs). **(H)** Fluorescent traces (grey lines) superimposed for the 20 frames (1.25 seconds) following the onset of the 36 presentations of the preferred visual stimulus for an example neuron. Red lines represent the positions of inferred spikes. The yellow line indicates the average fluorescence across all frames and presentations. **(I)** Heat map of ISR for all combinations of orientation and SF (in cpd) for a representative neuron (G, H). **(J)** Distribution of SNR values (black circles) for 2,114 ROIs from 8 P28 nondeprived mice. SNR is plotted (left) versus the frame with the optimal delay. The total number of neurons with an optical delay is plotted (right) versus the frame number. Frames 4–9 correspond to the optimal delay of time-locked neurons (red), while frame outside this range are spontaneously active (blue). **(K)** The SNR of spontaneously active neurons with optimal delays outside of frames 4–9 and the SNR of time-locked neurons with optical delays between frames 4–9 (J). The black vertical line indicates the threshold of the 75th percentile of the SNR of spontaneously active neurons. **(L)** The SR is the percent of presentations of the optimal visual stimulus at the frame of optimal delay for spontaneous and time-locked neurons in (J). The black line represents the threshold of the 75th percentile of the SR of spontaneously active neurons (with an optimum delay outside of frames 4–9). **(M)** Scatter plot of SNR versus SR for spontaneously active neurons. More than 96% are below the intersection of these criteria for visual responsiveness. **(N)** Scatter plot of SNR and percent responses for time-locked neurons. (**O)** Panel J replotted with the visually responsive neurons (red) and spontaneously active neurons (blue) indicated. The dashed line represents the average number of spontaneously active neurons with optimal delays at outside of frames 4–9. Note that the number of spontaneously active neurons between frames 4–9 equals or exceeds that of the surrounding frames. The y-axis is shown on the right to match panel (J). https://data.mendeley.com/datasets/3yt5kpzw6d. cpd, cycles per degree; ISR, inferred spike rate; ROI, region of interest; SF, spatial frequency; SNR, signal-to-noise ratio; SR, spike ratio.
(TIF)

S2 Fig The effects of MD on preferred orientation and preferred SF tuning properties of neurons for mice during the critical period. **(A)** Heat map of neuronal orientation preference for the contralateral eye for the P28 neurons presented in Fig 1A. Scale bar equals 100 μm. **(B)** Heat map of neuronal SF preference for the contralateral eye for the P28 neurons presented in Fig 1A. Scale bar equals 100 μm. **(C)** Preferred orientation for the contralateral eye (C) and ipsilateral eye (I) for all nondeprived P28-P32 mice in Fig 1. **(D)** Difference in the preferred orientation for the contralateral eye and ipsilateral eye by binocular neurons (*n =* 474) plotted against the preferred SF for the contralateral eye for nondeprived P28-P32 mice. **(E)** Preferred orientation plotted against preferred SF for the contralateral eye for neurons (*n* = 1,566) from nondeprived P28-P32 mice. **(F)** Preferred SF for the ipsilateral eye plotted against preferred SF for the contralateral eye for nondeprived P28-P32 mice. **(G)** Preferred orientation for the contralateral eye (C) and ipsilateral eye (I) for P32 mice receiving 4 days of MD to the contralateral eye (P32MD) in Fig 1. **(H)** Difference in the preferred orientation for the contralateral eye and ipsilateral eye by binocular neurons (*n* = 162) plotted against the preferred SF for the contralateral eye for P32 4-day MD mice. **(I)** Preferred orientation plotted against preferred SF for the contralateral eye for P32 4-day MD mice (*n* = 447). **(J)** Preferred SF for the ipsilateral eye plotted against preferred SF for the contralateral eye for P32 4-day MD mice. https://data.mendeley.com/datasets/3yt5kpzw6d. MD, monocular deprivation; SF, spatial frequency.
(TIF)

S3 Fig Repeated calcium imaging of neurons in V1 to measure the stability of tuning properties and the neuronal composition of visual circuitry. **(A)** Example reference images for the imaging plane of neurons at P28 (left) and P32 after 4 days of MD (right). Scale bar = 100 μm. Landmarks of strongly responding neurons (gold filled arrowheads) and features of the microvasculature (gold open arrowheads) are used to identify the same location. **(B)** Populations of segmented ROIs at P28 (green outlines) and P32MD (red outlines). ROIs with at least 50% overlap are filled (yellow). A perimeter of overlapping ROIs subsequently determined to be visually responsive at both time points and possess an orientation preference that differs by less than 30 degrees and SF preference that differs by less than 1 octave are circled (white outline). These neurons define region the analysis. **(C)** The preferred orientation of perimeter neurons at P28 and P32MD. Black lines connect pairs. **(D)** The preferred SF of perimeter neurons at P28 and P32MD. Black lines connect pairs. **(E)** Difference in the preferred orientation of perimeter neurons at P28 and P32MD. **(F)** Difference in the preferred SF of perimeter neurons at P28 and P32MD. **(G)** The P32MD/P28 SNR ratio of neurons, which were visually responsive at both P28 and P32MD (stable), neurons that were visually responsive at P28 but not P32MD, and neurons that were not visually responsive at P28 but were visually responsive at P32MD. (Kruskal–Wallis test with Dunn’s correction). **(H)** The P32MD/P28 SR of neurons, which were visually responsive at both P28 and P32MD (stable), neurons that were visually responsive at P28 but not P32MD, and neurons that were not visually responsive at P28 but were visually responsive at P32MD. (Kruskal–Wallis test with Dunn’s correction). https://data.mendeley.com/datasets/3yt5kpzw6d. MD, monocular deprivation; ROI, region of interest; SF, spatial frequency; SNR, signal-to-noise ratio; SR, spike ratio.
(TIF)

S4 Fig Comparisons of preferred orientation, matching of preferred orientation, and preferred SF for mice receiving 4 days of MD. **(A)** A scatter plot of the difference in preferred orientation (binocular matching) for neurons that were binocular both at P28 and after 4 days of MD (B-B, *n =* 102). **(B)** A scatter plot of binocular matching plotted against the difference in preferred orientation at P28 and P32MD for the contralateral eye (B-B, *n* = 102). (**C)** A scatter plot of binocular matching plotted against the difference in preferred orientation at P28 and P32MD for the ipsilateral eye (B-B, *n* = 102). **(D)** A scatter plot of binocular matching difference for neurons that interconverted to binocular at P32 following MD from monocular contralateral (red) (C-B, *n* = 87) and monocular ipsilateral (blue) at P28 (I-B, *n* = 36) plotted against the difference in preferred orientation at P28 and P32MD. **(E)** The difference in preferred orientation at P28 and P32MD for contralateral monocular neurons (C-B, red) and ipsilateral monocular neurons (I-B, blue) that converted to binocular neurons at P32 after 4 days of MD from panels D and E. **(F)** The difference in preferred orientation for neurons that were monocular contralateral (red, *n* = 95), binocular (green, *n* = 102), or monocular ipsilateral (blue, *n* = 39) at both P28 and P32 after 4 days of MD. The preferred orientations for the contralateral eye and ipsilateral eye are shown separately for binocular neurons (B-C and B-I, respectively). https://data.mendeley.com/datasets/3yt5kpzw6d. MD, monocular deprivation; SF, spatial frequency.
(TIF)

## Abbreviations

cpd: cycles per degree
ISR: inferred spike rate
L: layer
MD: monocular deprivation
OD: ocular dominance
ODI: ocular dominance index
P: postnatal day
ROI: region of interest
SF: spatial frequency
SNR: signal-to-noise ratio
SR: spike ratio
TTL: transistor–transistor logic
VEP: visually evoked potential
