# Supplementary figures and images for: Monocular deprivation during the critical period alters neuronal tuning and the composition of visual circuitry
Source: PLoS Biol. 2023 Apr 21;21(4):e3002096. doi: 10.1371/journal.pbio.3002096 (PMC10155990; doi:10.1371/journal.pbio.3002096)

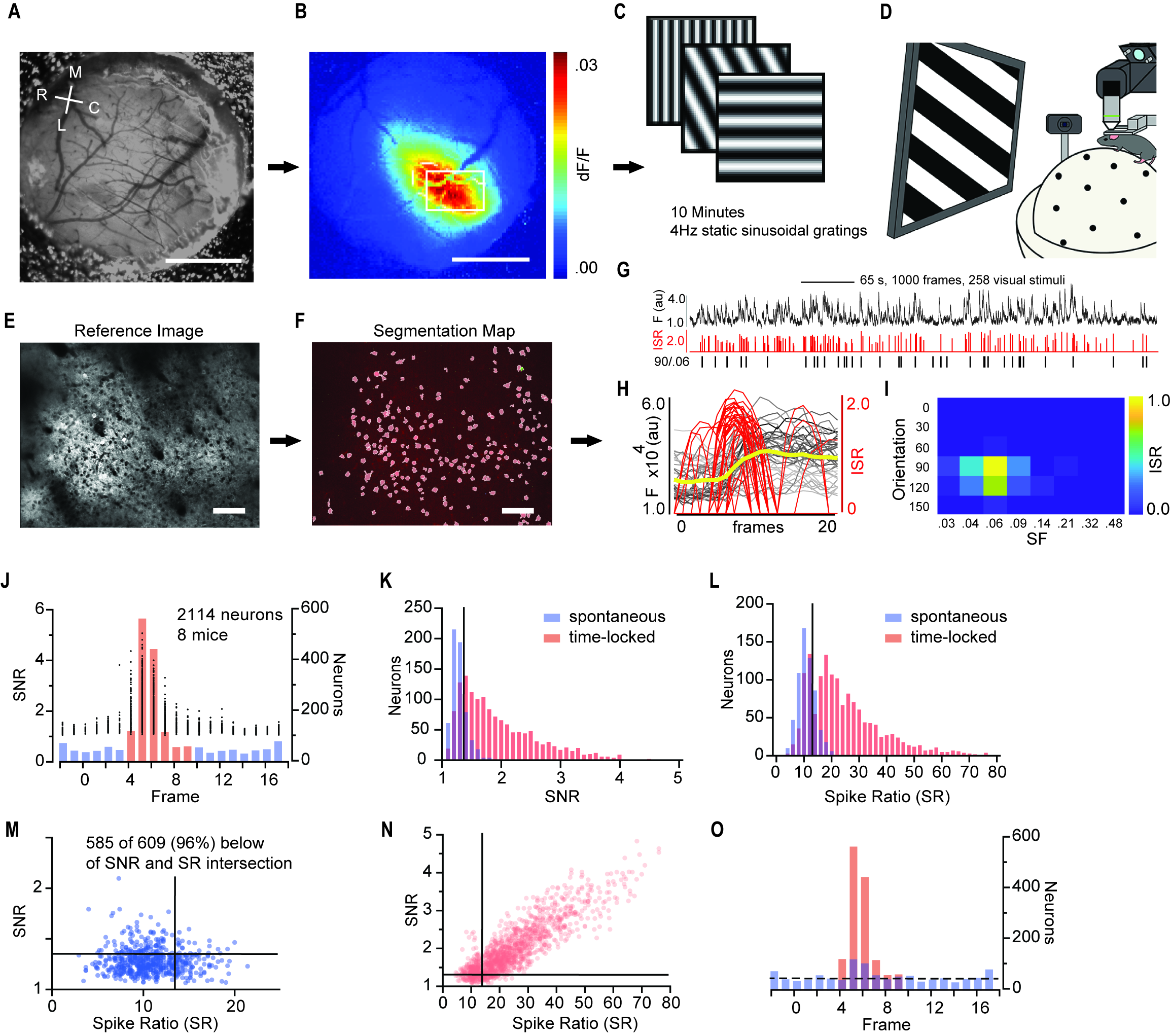

Supplement: S1 Fig — (A) A cranial window 3 mm in diameter implanted over visual cortex. Scale bar = 1 cm. Rostral–caudal and medial–lateral axes are indicated at upper left. (B) Wide-field calcium imaging of neuronal activity in response to a horizonal bar 30 degrees wide and 2 degrees high drifting down at 10 degrees per second. The white rectangle indicates the imaging field in (C) and (D). Scale bar = 1 cm. (C) Schematic of the visual stimulus. Sinusoidal gratings at 30 degrees intervals in orientation and between 0.028 and 0.48 cpd in SF spaced at half octaves (log(1.5)) as well as an isoluminant grey screen are presented in random order at 4 Hz for 10 minutes. Each combination of orientation and SF is presented 40 times on average (range 29–56). (D) Schematic of the setup for calcium imaging of alert mice. The monitor is positioned 35 cm away from the mouse centered at the zero azimuth and elevation. A mouse is alert, head-fixed, and freely moving on a styrofoam ball floating on column of air. A camera records pupil diameter. (E) An example reference image of imaging plane in the binocular zone of visual cortex. Imaging field is 750 μm × 500 μm. Scale bar = 100 μm. (F) Segmented neurons from the imaging field in (E). White circles correspond to ROIs identified manually. A total of 215 neuronal ROIs are segmented in this field. Scale bar = 100 μm. (G) Representative calcium trace (black line, top) and ISR (red line, middle) from an example neuron in the imaging field in (F). The timing of presentations of the preferred stimulus (90 degrees, 0.06 cpd) during the experiment (black vertical lines, bottom). Images are collected at 15.5 Hz. The scale bar represents 65 seconds, 1,000 frames, and 258 visual stimuli (grey horizonal bar, top right). Entire trace represents 10 minutes during which 2,400 gratings were presented from 56 combinations (6 orientations and a grey screen for each of 8 SFs). (H) Fluorescent traces (grey lines) superimposed for the 20 frames (1.25 seconds) following [file pbio.3002096.s001.tif]

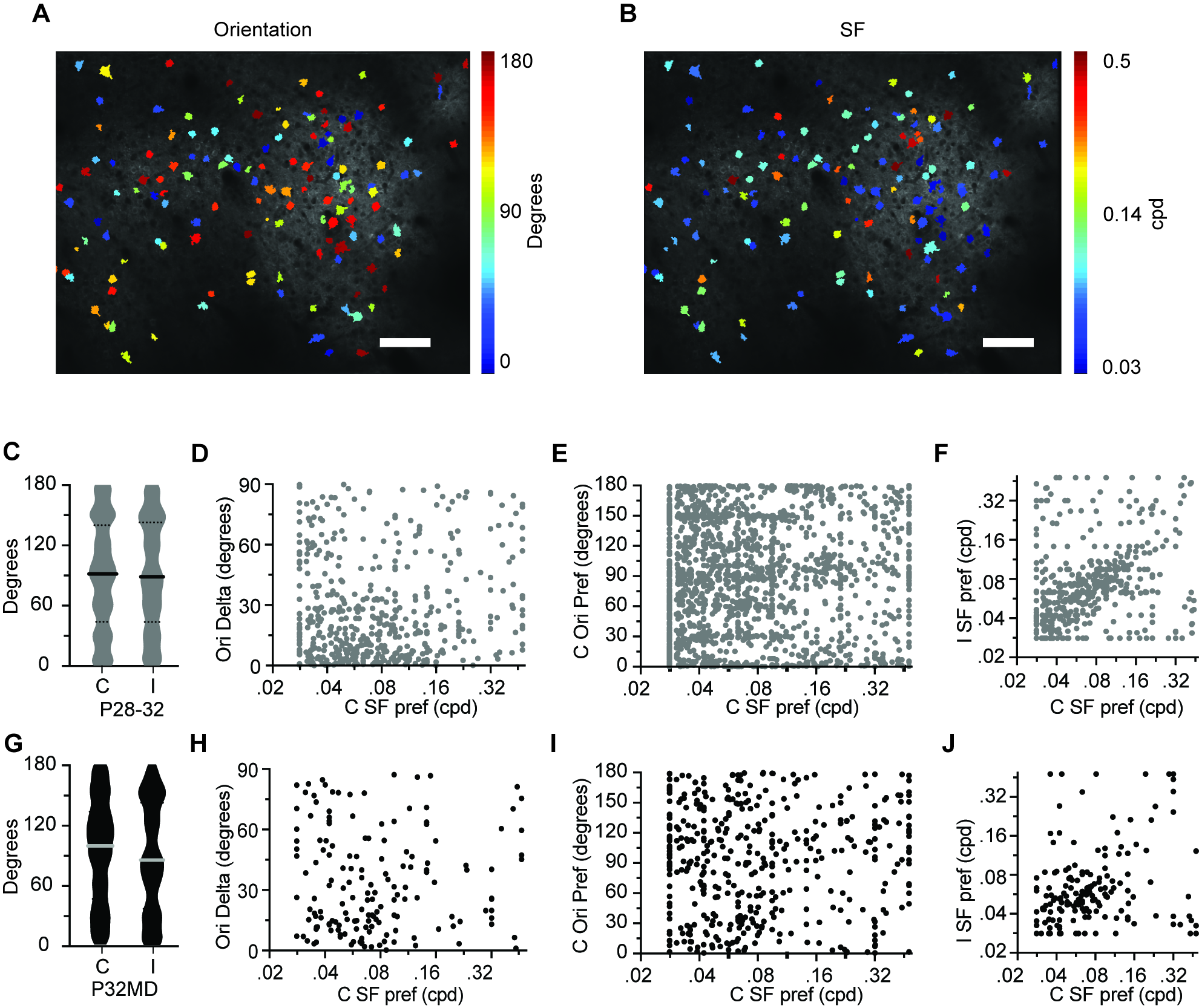

Supplement: S2 Fig — (A) Heat map of neuronal orientation preference for the contralateral eye for the P28 neurons presented in Fig 1A. Scale bar equals 100 μm. (B) Heat map of neuronal SF preference for the contralateral eye for the P28 neurons presented in Fig 1A. Scale bar equals 100 μm. (C) Preferred orientation for the contralateral eye (C) and ipsilateral eye (I) for all nondeprived P28-P32 mice in Fig 1. (D) Difference in the preferred orientation for the contralateral eye and ipsilateral eye by binocular neurons (n = 474) plotted against the preferred SF for the contralateral eye for nondeprived P28-P32 mice. (E) Preferred orientation plotted against preferred SF for the contralateral eye for neurons (n = 1,566) from nondeprived P28-P32 mice. (F) Preferred SF for the ipsilateral eye plotted against preferred SF for the contralateral eye for nondeprived P28-P32 mice. (G) Preferred orientation for the contralateral eye (C) and ipsilateral eye (I) for P32 mice receiving 4 days of MD to the contralateral eye (P32MD) in Fig 1. (H) Difference in the preferred orientation for the contralateral eye and ipsilateral eye by binocular neurons (n = 162) plotted against the preferred SF for the contralateral eye for P32 4-day MD mice. (I) Preferred orientation plotted against preferred SF for the contralateral eye for P32 4-day MD mice (n = 447). (J) Preferred SF for the ipsilateral eye plotted against preferred SF for the contralateral eye for P32 4-day MD mice. https://data.mendeley.com/datasets/3yt5kpzw6d. MD, monocular deprivation; SF, spatial frequency. (TIF) [file pbio.3002096.s002.tif]

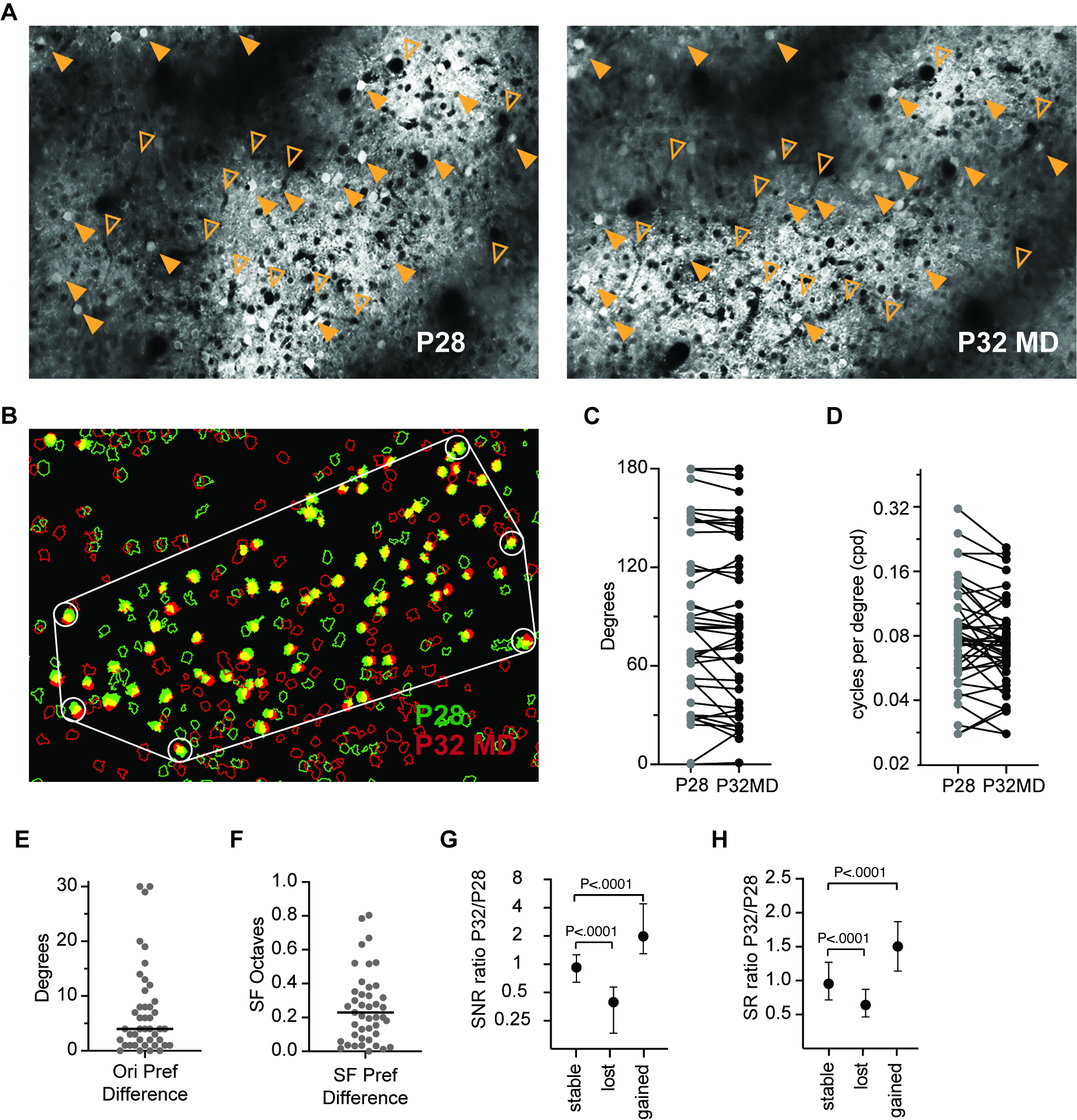

Supplement: S3 Fig — (A) Example reference images for the imaging plane of neurons at P28 (left) and P32 after 4 days of MD (right). Scale bar = 100 μm. Landmarks of strongly responding neurons (gold filled arrowheads) and features of the microvasculature (gold open arrowheads) are used to identify the same location. (B) Populations of segmented ROIs at P28 (green outlines) and P32MD (red outlines). ROIs with at least 50% overlap are filled (yellow). A perimeter of overlapping ROIs subsequently determined to be visually responsive at both time points and possess an orientation preference that differs by less than 30 degrees and SF preference that differs by less than 1 octave are circled (white outline). These neurons define region the analysis. (C) The preferred orientation of perimeter neurons at P28 and P32MD. Black lines connect pairs. (D) The preferred SF of perimeter neurons at P28 and P32MD. Black lines connect pairs. (E) Difference in the preferred orientation of perimeter neurons at P28 and P32MD. (F) Difference in the preferred SF of perimeter neurons at P28 and P32MD. (G) The P32MD/P28 SNR ratio of neurons, which were visually responsive at both P28 and P32MD (stable), neurons that were visually responsive at P28 but not P32MD, and neurons that were not visually responsive at P28 but were visually responsive at P32MD. (Kruskal–Wallis test with Dunn’s correction). (H) The P32MD/P28 SR of neurons, which were visually responsive at both P28 and P32MD (stable), neurons that were visually responsive at P28 but not P32MD, and neurons that were not visually responsive at P28 but were visually responsive at P32MD. (Kruskal–Wallis test with Dunn’s correction). https://data.mendeley.com/datasets/3yt5kpzw6d. MD, monocular deprivation; ROI, region of interest; SF, spatial frequency; SNR, signal-to-noise ratio; SR, spike ratio. (TIF) [file pbio.3002096.s003.tif]

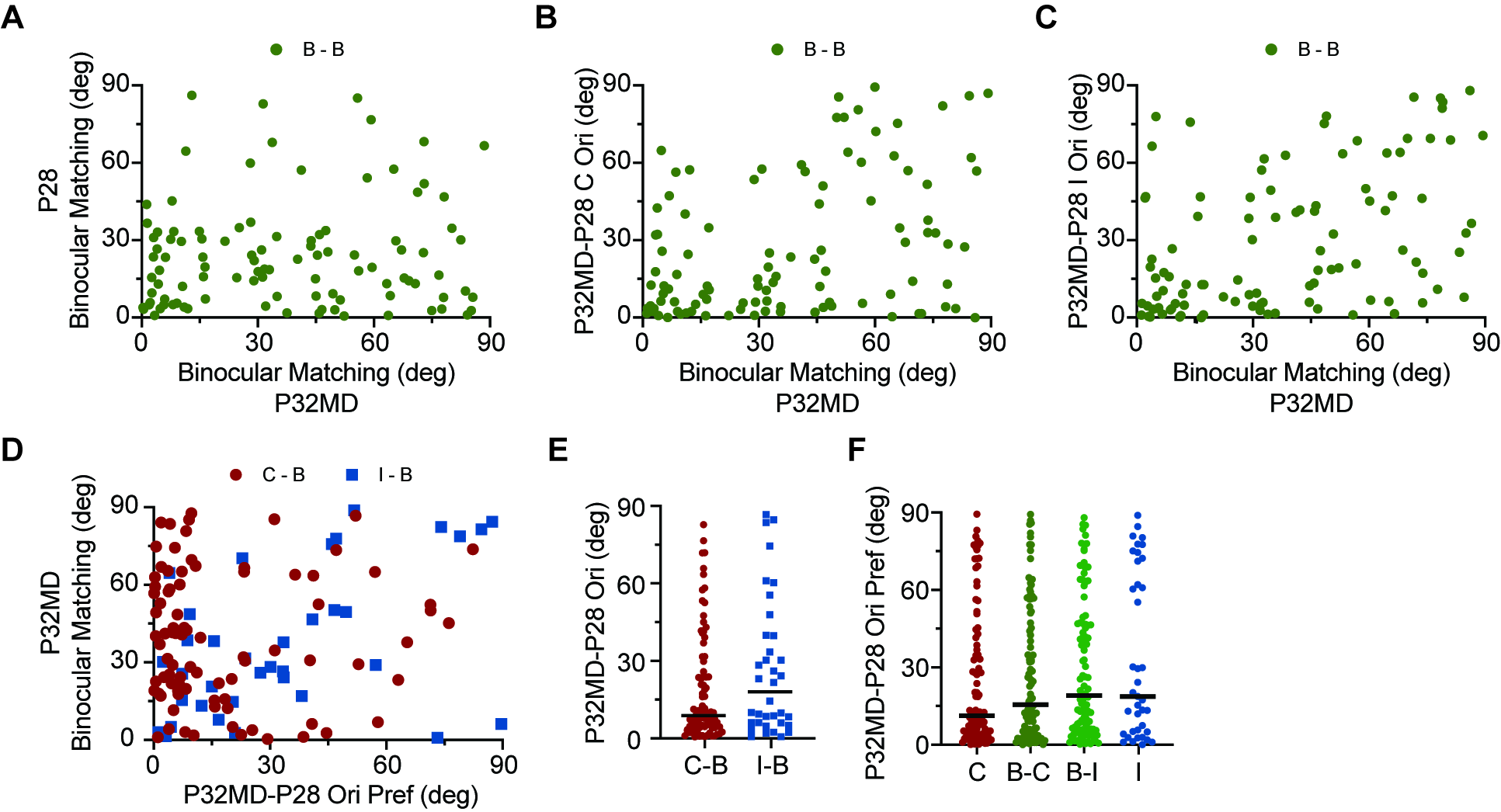

Supplement: S4 Fig — (A) A scatter plot of the difference in preferred orientation (binocular matching) for neurons that were binocular both at P28 and after 4 days of MD (B-B, n = 102). (B) A scatter plot of binocular matching plotted against the difference in preferred orientation at P28 and P32MD for the contralateral eye (B-B, n = 102). (C) A scatter plot of binocular matching plotted against the difference in preferred orientation at P28 and P32MD for the ipsilateral eye (B-B, n = 102). (D) A scatter plot of binocular matching difference for neurons that interconverted to binocular at P32 following MD from monocular contralateral (red) (C-B, n = 87) and monocular ipsilateral (blue) at P28 (I-B, n = 36) plotted against the difference in preferred orientation at P28 and P32MD. (E) The difference in preferred orientation at P28 and P32MD for contralateral monocular neurons (C-B, red) and ipsilateral monocular neurons (I-B, blue) that converted to binocular neurons at P32 after 4 days of MD from panels D and E. (F) The difference in preferred orientation for neurons that were monocular contralateral (red, n = 95), binocular (green, n = 102), or monocular ipsilateral (blue, n = 39) at both P28 and P32 after 4 days of MD. The preferred orientations for the contralateral eye and ipsilateral eye are shown separately for binocular neurons (B-C and B-I, respectively). https://data.mendeley.com/datasets/3yt5kpzw6d. MD, monocular deprivation; SF, spatial frequency. (TIF) [file pbio.3002096.s004.tif]
